# MTAGCN: predicting miRNA-target associations in *Camellia sinensis* var. assamica through graph convolution neural network

**DOI:** 10.1186/s12859-022-04819-3

**Published:** 2022-07-11

**Authors:** Haisong Feng, Ying Xiang, Xiaosong Wang, Wei Xue, Zhenyu Yue

**Affiliations:** School of Information and Computer, Anhui Provincial Engineering Laboratory for Beidou Precision Agriculture Information, Anhui Agricultural University, Hefei, 230036 Anhui China

**Keywords:** CSA miRNA-target association prediction, Deep learning, Graph convolution network, Layer attention mechanism

## Abstract

**Background:**

MircoRNAs (miRNAs) play a central role in diverse biological processes of *Camellia sinensis* var.assamica (CSA) through their associations with target mRNAs, including CSA growth, development and stress response. However, although the experiment methods of CSA miRNA-target identifications are costly and time-consuming, few computational methods have been developed to tackle the CSA miRNA-target association prediction problem.

**Results:**

In this paper, we constructed a heterogeneous network for CSA miRNA and targets by integrating rich biological information, including a miRNA similarity network, a target similarity network, and a miRNA-target association network. We then proposed a deep learning framework of graph convolution networks with layer attention mechanism, named MTAGCN. In particular, MTAGCN uses the attention mechanism to combine embeddings of multiple graph convolution layers, employing the integrated embedding to score the unobserved CSA miRNA-target associations.

**Discussion:**

Comprehensive experiment results on two tasks (balanced task and unbalanced task) demonstrated that our proposed model achieved better performance than the classic machine learning and existing graph convolution network-based methods. The analysis of these results could offer valuable information for understanding complex CSA miRNA-target association mechanisms and would make a contribution to precision plant breeding.

**Supplementary Information:**

The online version contains supplementary material available at 10.1186/s12859-022-04819-3.

## Introduction

Tea, produced from the dried leaves of tea plant, *Camellia sinensis*, is one of the most widely consumed drink in the world, which has large economic, medicinal and cultural significance [1]. Many studies demonstrated that the characteristic secondary metabolites in tea leaves such as polyphenols, caffeine, theanine, vitamins, have numerous health and medical benefits for humans [2, 3]. Plant microRNAs (miRNAs) are highly conserved and play an important role in gene expression regulation by targeting specific mRNA [4]. Furthermore, it is proven that miRNAs are involved in the development procedures, stress responses or biosynthesis of the secondary metabolites in *Camellia sinensis* var.assamica (CSA) [5, 6]. Thus, the identification of CSA miRNAs can not only improve the understanding of miRNA targeted gene regulation but also the evolution of miRNAs.

Although experiment methods to identifying CSA miRNA-target have high accuracy [7], they may suffer from time-consuming, laborious and expensive. As a result, it is necessary to develop computational methods for predicting miRNA-target association. Machine learning or deep learning-based methods have been generally adopted to solve various association pair prediction problems in biology. For example, many classification algorithms regard the associations as samples firstly, and the feature vectors of the edges are used to represent these samples. Then the classifiers are trained to recognize the real-existing associations in the graph [8, 9]. Nevertheless, the above machine learning methods are heavily dependent on the negative data sampling and the feature extraction. Therefore, more advanced machine learning methods, such as label propagation [10], regularized least squares [11], semi-supervised graph cut [12], sparse subspace learning [13], matrix factorization [14] and matrix completion [15, 16], are introduced to solve these kinds of problems. Matrix completion and matrix factorization methods are popular in community due to their flexibility in aggregating apriori information [17]. However, deploying them on high-dimensionality data is challenging because of the high computational complexity of matrix operations.

Deep learning methods have recently shown excellent performance in many fields, such as perception, planning, localization, and control [18]. The excellent capabilities of deep learning methods for learning representations from the complicated data make it extremely suitable for predicting association pairs in biology. Graph neural network (GNN) uses different node neighborhood aggregating schemes, representing a significant progress in directly processing network/graph structure data [19]. Each node feature can be updated by aggregating features of its neighboring nodes during the layer propagation and the node embedding will naturally capture the graph structure. GNNs have been extensively applied in multifarious problems, achieving superior performance in biological tasks, such as disease-gene association identification [20, 21], drug-drug interaction predictions [22, 23], miRNA-disease association predictions [24, 25], etc. As an extension of convolutional neural network for processing graph data, graph convolution network (GCN) [26], an important branch of GNN, has achieved excellent performance in different tasks. It is an end-to-end architecture and captures the graph structural information through messages passed between graph nodes, thereby retains explainability. In recent years, it shows superior performances in biological network analysis [27, 28].

In this paper, we developed a graph convolutional network model (MTAGCN) for predicting CSA miRNA-target associations. At first, we constructed heterogeneous networks by exploiting the CSA miRNA-target associations, miRNA-miRNA similarity matrix and target-target similarity matrix. Next, the graph convolution operation was conducted on the heterogeneous network to learn CSA miRNAs and targets embeddings. Considering that the embeddings from different convolution layers represent the proximity of nodes in the network at different levels [29], we introduced the attention mechanism [30] to combine useful neighborhoods representation adaptively and dynamically. Finally, we defined a score function which based on the integrated embedding, giving predictive scores for unobserved miRNA-target associations. Comprehensive experiment results on two tasks, i.e. the balanced task and the unbalanced task, showed that our proposed MTAGCN model had a better performance than five machine learning and three existing state-of-the-art methods.

In summary, our main contributions are as follows:

- We constructed the heterogeneous network to effectively integrate rich biological information, including CSA miRNA-target associations, CSA miRNA information and CSA target information.
- We proposed MTAGCN, a novel GCN-based method for predicting CSA miRNA-target associations. To our knowledge, this is the first work to adapt deep learning method for CSA miRNA-target association prediction.
- We designed the attention mechanism to integrate the embeddings information from multiple convolutional layers, leading to more useful representation from miRNAs and targets.

## Methods and materials

### Data

The data we used in this study was collected from the 2020 version of the CSA miRNA-target associations released in the work of Suo et al. [7]. This dataset contains 5264 relationships between CSA miRNAs and targets which include 356 miRNAs and 4041 targets. For the lack of some miRNA sequences and target information, we removed the relationships between miRNAs and targets, including 66 miRNAs and 1166 targets. Therefore, the resulting dataset we obtained contains 3745 miRNA-target pairs, including 290 different types of CSA miRNAs and 2876 targets. Then, we acquired the CSA target gene locations from http://teacon.wchoda.com, a database of gene co-expression network for CSA plant [31]. According to the CSA target gene locations, we extracted target sequences from CSA whole genome data in the Tea Plant Information Archive [32]. The details are shown in Table 1.

**Table 1:** Summary of the statistics of the miRNA-target associations

Data	MiRNA	Target	Association
Original	356	4041	5264
Used	290	2876	3745

Original, the original data from the work of *Suo *et al., Used, the data after filtering

To perform the five-fold cross validation, we developed a balanced and an unbalanced dataset, respectively, to evaluate the CSA miRNA-target prediction models. In the training dataset, four-fifths of the positive samples and all the negative samples are used. As for test set in the unbalanced task, we use the remaining one-fifth of the positive samples (749 positive samples) and draw 20 times number of positive samples as negative samples (14,980 negative samples). Then the class of negative data vastly outnumbers that of positive data, causing a class imbalance problem (Table 2). As for test set in the balanced task, we used the same number of negative samples as positive samples (Table 2). In addition, in order to acquire CSA miRNA similarities and target similarities, Kmer [33], an algorithm based on nucleic acid composition, is used to transform the CSA targets sequences and miRNAs sequences into feature vectors.

**Table 2:** Summary of the samples on the balanced and unbalanced data

Data	Training		Test	
	Positive	Negative	Positive	Negative
Balanced	2996	All	749	749
Unbalanced	2996	All	749	14,980

All, all miRNA-target pairs except for the positive samples

### Construction of heterogeneous network

#### Construction of similarity network

As mentioned above, we used Kmer to obtain CSA miRNA and target features. For one miRNA or target binary feature vector, each element means whether the feature descriptor is present or absent. In this work, we adopted the Jaccard index to calculate the miRNA-miRNA and target-target similarities. Jaccard index [27] is a prevailing measure for calculating similarity based on these features. Thus, we further constructed miRNA similarity matrix and target similarity matrix. The Jaccard index measure between two vectors $x_{i}$ and $x_{j}$ is defined as follows:

1 $$S_{ij}^{r} = \frac{{\left| {x_{i} \cap x_{j} } \right|}}{{\left| {x_{i} \cup x_{j} } \right|}}$$

where $\left| {x_{i} \cap x_{j} } \right|$ denotes the number of features where both elements in $x_{i}$ and the related ones of $x_{j}$ equal to 1, and $\left| {x_{i} \cup x_{j} } \right|$ denotes the numbers of features where either the elements of $x_{i}$ or the related ones of $x_{j}$ equal to 1.

Herein, we also considered other similarity calculation measures to construct similarity network, including cosine similarity, Gaussian kernel-based similarity, and Pearson similarity. These measures are widely used in constructing similarity network and have achieved great performance in many biological prediction tasks [22, 34, 35].

#### Heterogeneous network for CSA miRNAs and targets

The heterogeneous network is constructed based on miRNA-target associations, miRNA-miRNA similarity and target-target similarity.

The miRNA-target associations are denoted as an adjacent matrix ${ }A \in \left\{ {0,1} \right\}^{M*N}$, *M* and *N* represent the number of miRNAs and targets, respectively. If a CSA miRNA $r_{i}$ is associated with a target $t_{j}$, $A_{ij}$ = 1; otherwise ${ }A_{ij}$ = 0. The miRNA-miRNA similarity network is derived from the CSA miRNA similarity matrix $S^{m}$ with $S_{ij}^{m}$ as its (*i,j*)th element. And the target-target similarity network is derived from the CSA target similarity network $S^{n}$ with $S_{ij}^{n}$ as its (*i, j*)th element. Furthermore, we adapt $\sim S^{m} = D_{m}^{{ - \frac{1}{2}}} S^{m} D_{m}^{{ - \frac{1}{2}}}$ and $\sim S^{n} = D_{n}^{{ - \frac{1}{2}}} S^{n} D_{n}^{{ - \frac{1}{2}}}$ to normalize the similarity matrices, where $D_{m} = {\text{diag}}\left( {\mathop \sum \limits_{j} S_{ij}^{m} } \right)$ and $D_{n} = {\text{diag}}\left( {\mathop \sum \limits_{j} S_{ij}^{n} } \right)$. Finally, the heterogeneous network defined by the adjacency matrix comes to be

2 $$A_{H} = \left[ {\begin{array}{*{20}l} {\sim S^{m} } \hfill & A \hfill \\ {A^{T} } \hfill & {\sim S^{n} } \hfill \\ \end{array} } \right]$$

### Graph convolution

Regarding known associations between miRNAs and targets as a bipartite graph, the prediction problem in this paper can be defined as a semi-supervised link prediction task on such a graph.

We assume that a bipartite graph *G* = (*ν, ε*) with *ν* = **(**$\nu_{m, } \nu_{t} )$ including $n_{m }$ miRNA nodes and $n_{t}$ target nodes, which have numerical features $X_{m } = \left[ {x_{m}^{1} ,x_{m }^{2} , \ldots ,x_{m}^{{n_{m} }} } \right]^{T} \in R^{{n_{m} *M}}$ and $X_{t } = \left[ {x_{t}^{1} ,x_{t }^{2} , \ldots ,x_{t}^{{n_{t} }} } \right]^{T} \in R^{{n_{t} *N}}$, respectively. Supposing that partial links (denoted as *ε* in *G*) are given labels, our goal is to predict whether there are any potential links between miRNA and target that have not been determined previously. Thus, how best to effectively utilize both graph topology and the attribute information of the nodes is a problem we need to address.

There have recently been some attempts to use deep learning techniques to graph-based data analyses. A graph convolutional network (GCN) is proposed in Kipf et al. [26]. Graph convolution is defined on graph as the multiplication of an input signal with a filter $g_{\theta }$ in the Fourier domain [19]. Given an adjacent matrix *A* with its Laplacian *L*: *D-A*, and attributes of each node on graph (denoted as *s*), spectral graph convolution tries to decompose **s** on the spectral components. We assume that *L* can be decomposed by ${ }L = U\Lambda U^{T}$, *U* is eigenvector matrix and *Λ* is the diagonal matrix. Hence, $g_{\theta } {\text{*s}} = Ug_{\theta } U^{T}$ s is a graph Fourier transform of $U^{T} s$. Defferrard used a truncated expansion in terms of Chebyshev polynomials [36]$T_{k\left( s \right)}$ up to $K^{th}$ order, approximating the spectral filter in order to avoid the issue of computationally costly eigende-composition of *L*

3 $$g_{\theta } {\text{*s}} \approx { }\mathop \sum \limits_{k = 0}^{K} \theta^{\prime}_{k} T_{k} \left( {L_{N} } \right)s$$

where $\theta^{\prime}$ is a vector about Chebyshev coefficients and $T_{k}$ is the Chebyshev polynomials. A further research simplified this definition by approximating the largest eigenvalue of *L* by Formula (4) [26]. The convolution operator is

4 $$g_{\theta } {\text{*s}} = \theta \left( {{\text{I}} + D^{{ - \frac{1}{2 }}} {\text{A}}D^{{ - \frac{1}{2 }}} } \right)s$$

### Prediction framework of the proposed MTAGCN

**Fig. 1:**
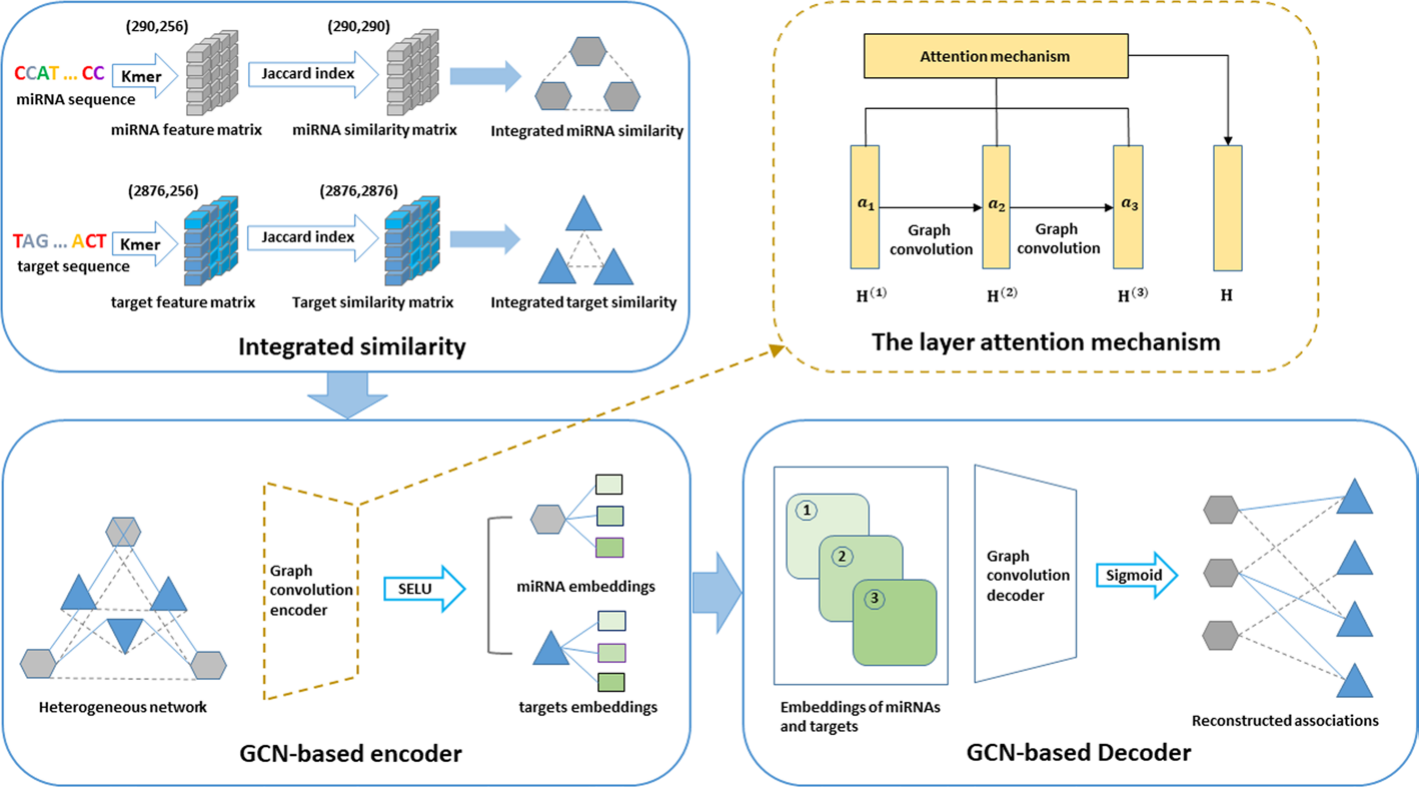
The workflow of MTAGCN for CSA miRNA-target association prediction

The workflow of our model is shown in Fig. 1. Our proposed MATGCN model consists of three parts, i.e., similarity network integration, encoder construction and decoder construction. We integrate similarity networks by combining rich biological information to construct the heterogeneous network. And the encoder is a GCN model with layer attention mechanism, capturing network structure information using GCN. We design a decoder, a fully connected layer network, to transform features into the original space.

For graph convolution, we adopted the simplified definition. As mentioned above, the prediction of associations between CSA miRNAs and targets can be considered as a semi-supervised link prediction problem. But current GCN-based approaches tackle node classification problem on homogeneous network and are not applicable to the issue involving prediction of associations. Thus, we extend the current graph convolution idea to solve link prediction problem defined on heterogeneous, bipartite, attributed networks. For this goal, we proposed the GCN-based framework called MTAGCN to solve the novel prediction problem. The Algorithm 1 shows the detailed training steps of the MTAGCN for predicting CSA miRNA-target association.

GCN is a multilayer connected neural network and its propagation rule is defined as follows:

5 $$H^{{\left( {l + 1} \right)}} = \sigma \left( {D^{{ - \frac{1}{2 }}} AD^{{ - \frac{1}{2 }}} H^{\left( l \right)} W^{\left( l \right)} } \right) = f\left( {H^{\left( l \right)} ,G} \right)$$

6 $$D = {\text{diag}}\left( {\mathop \sum \limits_{j} G_{ij} } \right)$$

where *σ* is an adjustable activation function, *D* is the diagonal degree matrix, *A* is the adjacency matrix, $H^{\left( l \right)}$ is the nodes embedding in the *l*th layer and $W^{\left( l \right)}$ is the layer-wise trainable weight.

For constructing the encoder of MTAGCN, we consider how to fully use the CSA miRNA-miRNA similarity network, the CSA target-target similarity network and the miRNA-target associations through graph convolution network on the heterogeneous graph $A_{H}$. Specifically, we set the input graph *G* as

7 $$G = \left[ {\begin{array}{*{20}l} {\mu \sim S^{m} } \hfill & A \hfill \\ {A^{T} } \hfill & {\mu \sim S^{t} } \hfill \\ \end{array} } \right]$$

where *μ* is a penalty factor that controls the contribution of the similarity in MTAGCN’s propagation process,$S^{m}$ is the CSA miRNA-miRNA similarity matrix and $S^{n}$ is the target-target similarity matrix. To initialize embeddings, we introduce graph convolution into the latent factor model in the light of the nature ‘miRNA-target’ associations and the embedding matrix is reconstructed as

8 $$H^{\left( 0 \right)} = \left[ {\begin{array}{*{20}l} 0 \hfill & A \hfill \\ {A^{T} } \hfill & 0 \hfill \\ \end{array} } \right]$$

with the above setting, the MTAGCN encoder for first layer can be defined as

9 $$H^{\left( 1 \right)} = \sigma \left( {D^{{ - \frac{1}{2 }}} GD^{{ - \frac{1}{2 }}} H^{\left( 0 \right)} W^{\left( 0 \right)} } \right)$$

where $H^{\left( 1 \right)} \in {\text{R}}^{{\left( {M + N} \right)*k}}$ denotes the first-layer node embeddings in the heterogeneous matrix $A_{H}$, *k* is the embedding dimensionality and $W^{\left( 0 \right)} \in {\text{R}}^{{\left( {M + N} \right)*k}}$ is the trainable weight matrix of the first-layer. The MTAGCN encoders for subsequent layers follow the Formula (5) and *G* is defined in Formula (7). Herein, after *L* iteration, *L k*-dimensional CSA miRNA and target embeddings can be obtained. Furthermore, we introduce SELU (scaled exponential linear unit) [37] as the activation function used in MTAGCN graph convolution layers to accelerate learning procedure and enhance generalization performance.

Different layers of the embeddings capture different structural information. Such as, the first layer obtains direct edge information and other layers obtain the multi-hop neighbor information by iteratively updating the embeddings [38, 39]. Considering that different embeddings in different layers have various contributions, we introduce a self-attention mechanism, which adaptively combines embeddings and harvests final embeddings of CSA miRNAs and targets as $$\left[ {\begin{array}{*{20}c} {H_{I} } \\ {H_{G} } \\ \end{array} } \right] = \sum a_{l} H^{l}$$, where $H_{I} \in R^{{M{*}k}} { }$ is the final embeddings of miRNAs,${ }H_{G} \in R^{{N{*}k}}$ is the final embeddings of targets, $a_{l}$ is auto-learned by a single-layer feed-forward network.

To reconstruct adjacency matrix for CSA miRNA-target associations, a bilinear decoder $A^{\prime} = {\text{f}}\left( {H_{I} ,H_{G} } \right)$ is built as follows:

10 $$A^{{\prime }} = {\text{sigmoid}}\left( {H_{I} W^{{\prime }} H_{G}^{T} } \right)$$

where $W^{\prime} \in R^{k*k}$ is the trainable matrix. We denoted $A_{ij}^{^{\prime}} { }$ as the predicted scores of the CSA miRNA-target association, which is given by corresponding (*i*, *j*)th entry of $A^{\prime}.$
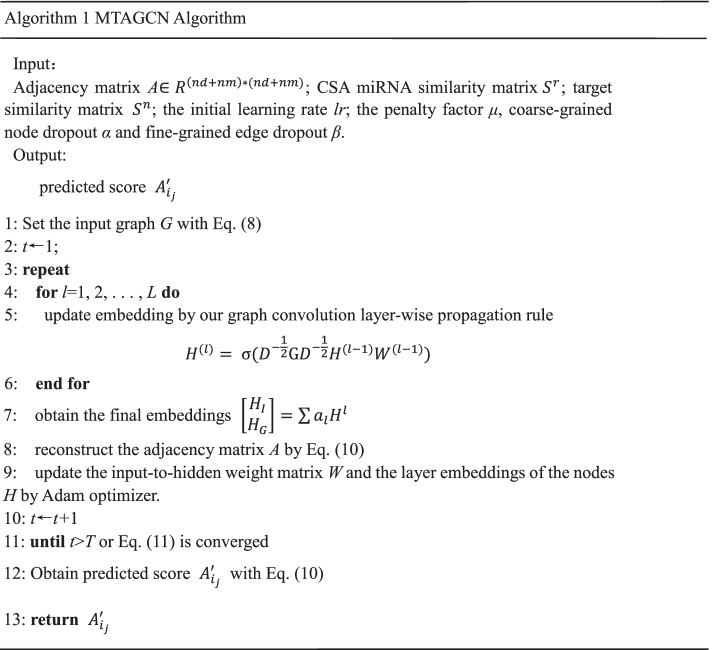


### Optimization

In the dataset with *M* CSA miRNAs and *N* targets, the miRNA-target association pairs are taken as the set of all positive association pairs $\gamma^{ + }$ and other pairs as the set of negative pairs $\gamma^{ - }$. Although it is a binary classification problem to differentiate two types of miRNA-target pairs, the number of negative miRNA–target pairs are much higher than that of the positive pairs. Herein, MTAGCN learns parameter by the loss function (weighted cross-entropy):

11 $${\text{loss}} = - \frac{1}{{\begin{array}{*{20}c} {M*N} \\ \end{array} }}\left( {\lambda {*}\mathop \sum \limits_{{\left( {i,j} \right) \in \gamma^{ + } }} \log A^{\prime}_{ij} + \mathop \sum \limits_{{\left( {i,j} \right) \in \gamma^{ - } }} \log \left( {1 - A_{ij}^{{\prime }} } \right)} \right)$$

where (*i, j*) is the instance for CSA miRNA $r_{i}$ and target $t_{j}$*,* $\lambda = \frac{{\left| {\gamma^{ - } } \right|}}{{\left| {\gamma^{ + } } \right|}}$, $\left| {\gamma^{ + } } \right|$ and $\left| {\gamma^{ - } } \right|$ denote the corresponding pairs. The balance factor *λ* emphasizes the known associations and decreases the impact of data imbalance.

The Xaiver initialization method [40] is used to randomly initialize all trainable weight matrices. Then, as is shown in the 9 rows of Algorithm 1, we use the Adam optimizer [41] for the optimization. In order to balance the training speed and the experimental result, we also use a simple cycle learning rate [42] during the optimization, that is making a change from 0.01 to 0.1. Furthermore, we introduce fine-grained edge dropout [43] and coarse-grained node dropout [44] in the graph convolution layers to prevent over-fitting. The fine-grained edge dropout is applied to convolution layers and dense layers, randomly drops out edges. And the coarse-grained node dropout can efficiently enforce dropout at the node level.
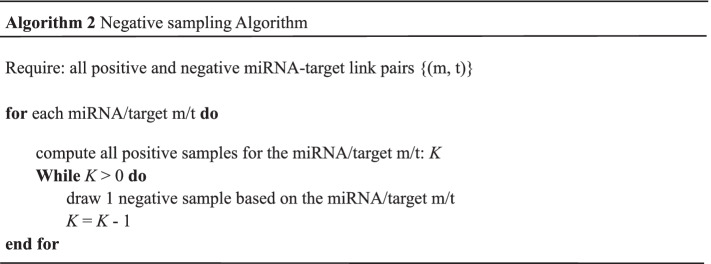


### Negative sampling

Recent arts usually focus on positive sampling, while the strategy for negative sampling is left insufficiently explored. However, many studies theoretically proved that negative sampling is important as positive sampling in determining the optimization objective and the resulted variance [45]. Hence, negative sampling has wide application in many fields for its simplicity and efficiency, such as natural language processing [46], computer vision [47], recommender system [48] and graph embedding [49]. Inspired by previous study [50], we adopted three strategies of negative sampling, including random negative sampling, sampling by CSA miRNA (SCM) and sampling by CSA target (SCT).

For random negative sampling, the negative samples were generated by randomly drawing from the total negative samples. Furthermore, we proposed two negative sampling methods, SCM and SCT. The two methods are similar in some ways and the details are shown in Algorithm 2. For SCM/SCT, we first computed all numbers of positive sample based on the per miRNA/target. Then the negative sample was drawn based on the corresponding miRNA/target. For the unbalanced task, we executed the SCM/SCT in a loop to get enough negative samples. Compared with the random negative sampling without regularity, the other two sampling methods can be based on one CSA miRNA or target. It is worth pointing out that SCM/SCT can ensure that every miRNA/target be sampled, greatly increasing the sampling range of negative samples. In the following, we will compare the above sampling strategies.

## Results and discussions

In this section, we briefly introduced the experimental setup. Next, we carried out to evaluate the performance of the proposed MTAGCN model and the effect of layer attention mechanism, then demonstrated the performance of our model by comparing with five machine learning methods and three existing link/association prediction methods on balanced and unbalanced tasks (Table 2), respectively.

### Experimental setting

To evaluate the effectiveness of our model, we performed five-fold cross validation on the two tasks. We randomly divided known miRNA-target associations into five subsets with equal size. For five-fold cross-validation, we randomly used the 80% known miRNA-target associations for training and the remaining 20% for test. We employed the AUPR (area under precision-recall curve) and the AUC (area under ROC curve) as primary metrics during cross validation which are widely used for pair-wise link predictions [51]. Besides, we also calculated other metrics, i.e. recall, specificity, precision, ACC and F1-score.

We set the embeddings dimensionality *k* as 64 by conducting the parameter sensitivity analysis. The layer number *L*, the initial learning rate *lr*, the coarse-grained node dropout *α* and the fine-grained edge dropout *β*, are respectively set to 3, 0.01, 0.6 and 0.6. In addition, the total training epochs of MTAGCN *γ* was set to 500, and the penalty factor *μ* was set to 0.06. Our experiment code was implanted on the open-source machine learning framework Tensorflow. All experiments were conducted on Ubuntu operating 20.04 system with a NVDIA GeForce GTX3090 GPU and 32G memory.

### The influence of different heterogeneous networks

MTAGCN takes advantage of the CSA miRNA-target heterogeneous network to construct the model. And we built the heterogeneous network by aggregating miRNA-miRNA similarities, target-target similarities and known miRNA-target associations. Since we took into account four similarity measures, MTAGCN could be trained on various heterogeneous networks, which may have a certain effect on the predictive ability.

MTAGCN models based on heterogeneous networks with different miRNA-miRNA and target-target similarities were evaluated by five-fold cross validation, and Table 3 shows the corresponding results. The Jaccard index achieved slightly better performance than the other similarity measures we used. And these results reflected that our model is robust. Based on the analysis, we ultimately employed Jaccard index to calculate CSA target-target similarity and CSA miRNA-miRNA similarity. In the following study, the heterogenous network was construct by fusing two similarity networks and CSA miRNA-target associations.

**Table 3:** Performances of MTAGCN based on different similarity measures

Measure	AUPR	AUC	F1	Accuracy	Recall	Specificity	Precision
Jaccard	**0.9207**	**0.8756**	**0.8555**	**0.8669**	**0.7886**	**0.9453**	**0.9356**
Cosine	0.8966	0.8805	0.8011	0.8212	0.7202	0.9222	0.9028
Pearson	0.8908	0.8699	0.7880	0.8123	0.7020	0.9226	0.9022
Gaussian	0.8360	0.7666	0.6928	0.7475	0.5733	0.9218	0.8788

The maximum value of each metric is bold

### Analysis of negative sampling

As mentioned above, we adopted three negative sampling strategies motivated by previous studies. We tested our model on these sampling strategies and then discussed how they influence the performances of MTAGCN. Table 4 shows that SCT achieves better results than both SCM and random negative sampling methods. That is maybe due to the number of targets is much more than that of miRNAs, resulting in a larger sampling range and reducing the sampling imbalance. To this end, we performed SCT strategy, setting the ratio of positive and negative samples to a rate of 1:1 (balanced task) and 1:20 (unbalanced task) in the follow-up test sets.

**Table 4:** Performances of MTAGCN based on different negative sampling strategies on the balanced task

Sampling	AUPR	AUC	F1	Accuracy	Recall	Specificity	Precision
SCT	**0.9207**	0.8756	**0.8555**	**0.8669**	0.7886	**0.9453**	**0.9356**
Random	0.8945	**0.8824**	0.7893	0.8110	0.7081	0.9140	0.8919
SCM	0.8914	0.8502	0.8332	0.8400	**0.8003**	0.8796	0.8695

The maximum value of each metric is bold. SCT, sampling by CSA target; Random, random negative sampling; SCM, sampling by CSA miRNA

### Results of MTAGCN

To develop the MTAGCN, we used the embeddings for diverse layers to construct models which denoted as MTAGCN-L1, MTAGCN-L2 and MTAGCN-L3. Table 5 shows the performance of the above models using five-fold cross validation. MTAGCN-L1 and MTAGCN-L2 performed better than MTAGCN-L3, showing that the lower layer captures more information than the higher layer because of the over-smoothing. However, MTAGCN that combines the embeddings for all three layers produced the best results on the balanced task.

**Table 5:** Performances of MTAGCN based on different embeddings for the balanced task

Performance	AUPR	AUC	F1	Accuracy	Recall	Specificity	Precision
Models							
MTAGCN	**0.9207**	**0.8756**	**0.8555**	**0.8669**	**0.7886**	0.9453	0.9356
MTAGCN-AVE	0.9134	0.8695	0.8208	0.8331	0.7648	0.9015	0.8863
MTAGCN-CON	0.9166	0.8657	0.8117	0.8348	0.7148	**0.9549**	**0.9416**
MTAGCN-L1	0.9052	0.8516	0.8347	0.8498	0.7583	0.9413	0.9284
MTAGCN-L2	0.8638	0.8288	0.7884	0.8084	0.7143	0.9025	0.8799
MTAGCN-L3	0.8499	0.8274	0.7409	0.7696	0.6617	0.8774	0.8438

The maximum value of each metric is bold

**Fig. 2:**
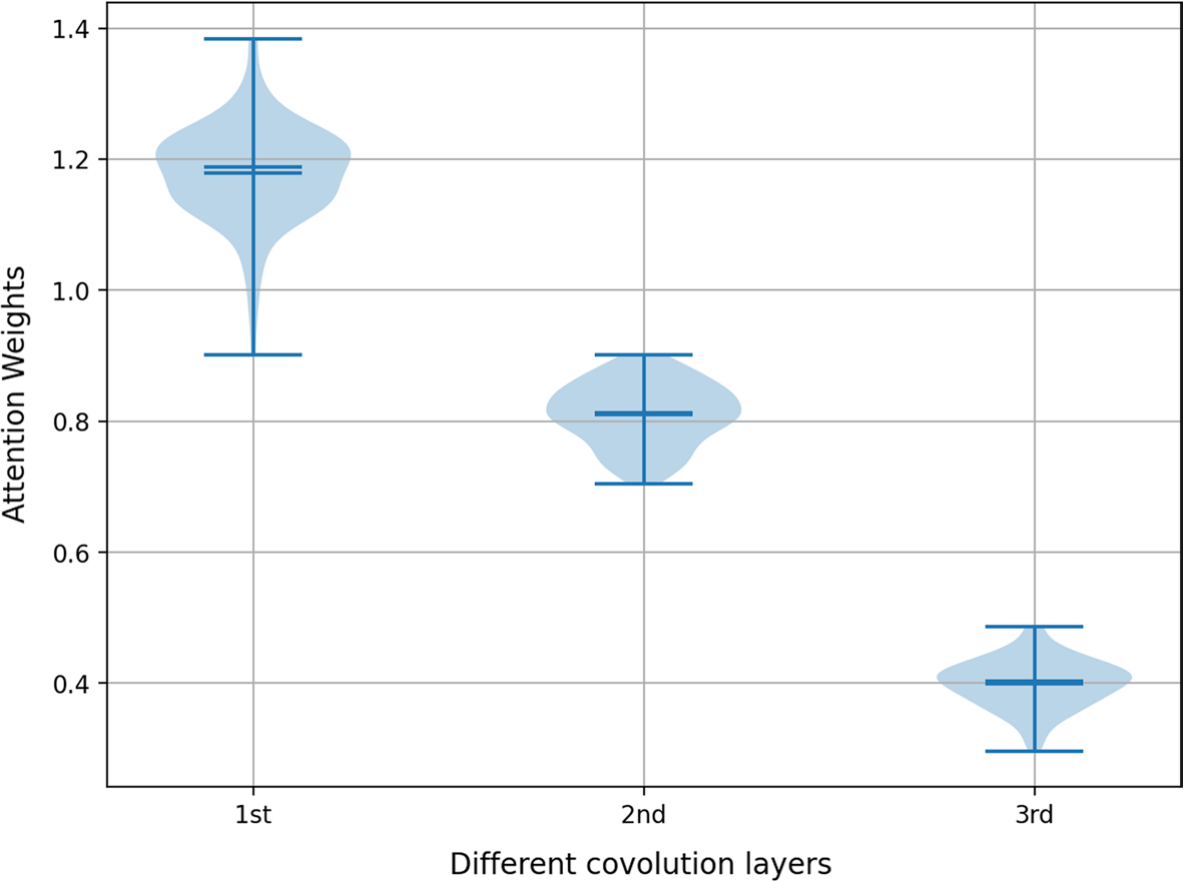
The attention weights of diverse convolution layers in MTAGCN

The *l*th layer of MTAGCN captures the *l*th-order proximity value between nodes, and the attention weights represent the relative contribution of the corresponding convolution layers. We implemented 20 runs of 5-cv, and the Fig. 2 visualizes the attention weights of diverse convolution layers. Different convolution layers have diverse weights, and that of the lower layer is greater than those of the higher layers, revealing that the lower-order proximity is of more important than the higher. Therefore, it also helps to illustrate the performance of MTAGCN-L1, MTAGCN-L2, MTAGCN-L3 (Table 5).

Furthermore, we considered MTAGCN-AVE and MTAGCN-CON, which integrate embeddings from different convolution layers. MTAGCN-AVE adopts the average of weights for different embeddings. As to MTAGCN-CON, we stack the embeddings for three layers directly. In Table 5, the results indicate that the MTAGCN with attention mechanism achieved more encouraging performance than MTAGCN-AVE and MTAGCN-CON. Additional file 1: Table S1 shows the results under unbalanced task, from which we can obtain similar conclusions.

### Comparison with the machine learning methods

**Fig. 3:**
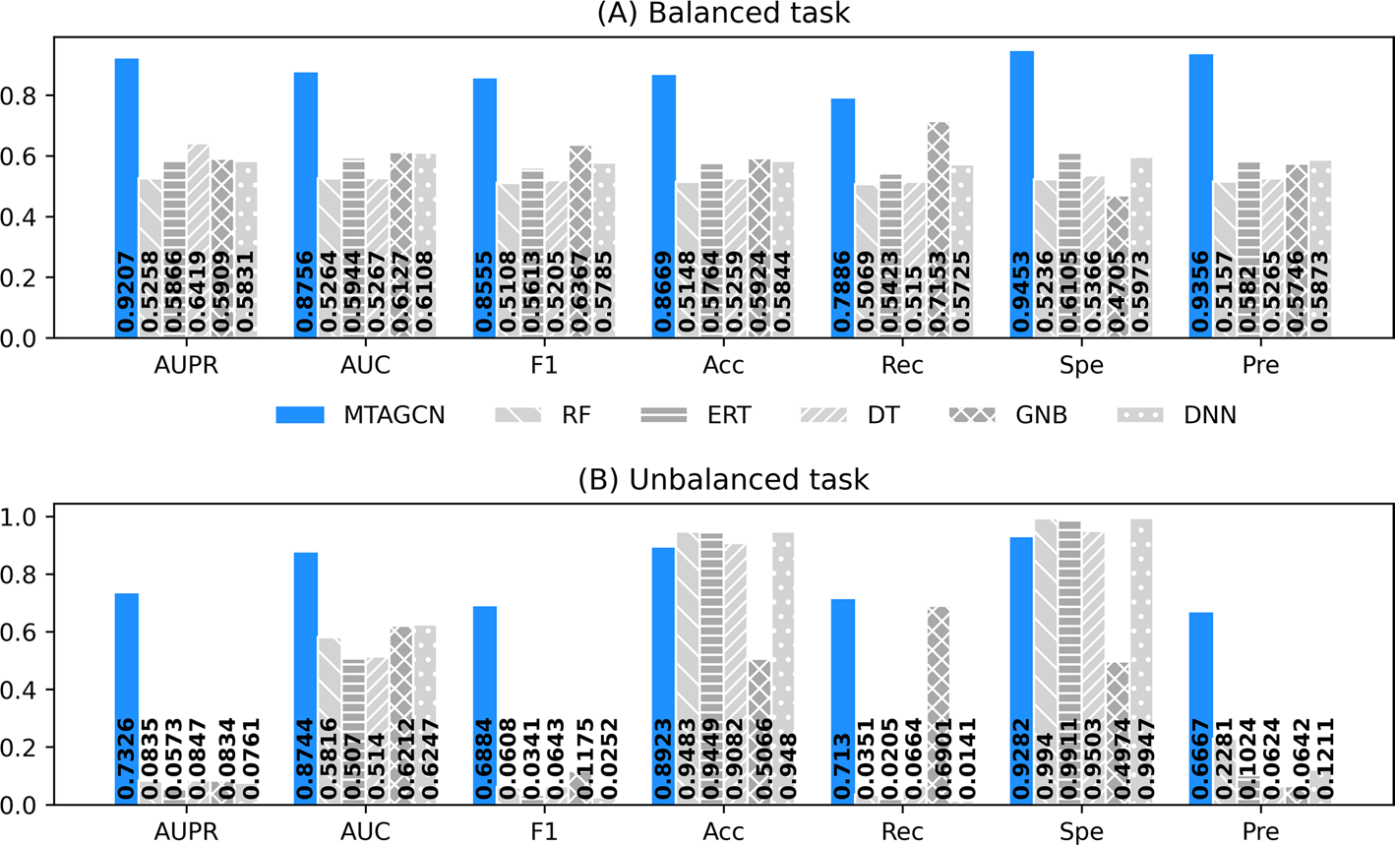
Performance comparison between MTAGCN and five types of classic machine learning methods on (**A**) the balanced task and (**B**) the unbalanced task, including RF (random forest), ERT (extremely randomized tree), DT (decision tree), GNBs (Gaussian naïve Bayes), DNN (deep neural network). F1, Acc, Rec, Spe and Pre represent the F1-score, accuracy, recall, specificity and precision, respectively

To investigate the performance of our proposed MTAGCN model for CSA miRNA-target association prediction, we compared it with some classic machine learning algorithms, including random forest (RF), extremely randomized tree (ERT), decision tree (DT), Gaussian naïve Bayes (GNBS), deep neural network (DNN). The results for the above machine learning models on the balanced and unbalanced task are shown in Fig. 3.

According to the results, MTAGCN outperforms all classic machine learning methods, strikingly for the balanced task in Fig. 3 (A). As to the unbalanced task, the accuracy and the specificity of the MTAGCN are lower than most of the classic machine learning models, but the primary metrics AUPR and AUC are higher than these models. It is believed that both the accuracy and specificity are threshold-based metrics, which are greatly affected by data imbalance [52]. Overall, MTAGCN has a better performance than the methods used. These classic machine methods all have a low AUPR, F1, and recall, which means the proposed model produces more robust performances across two tasks.

### Comparison with the state-of-the-art methods

As mentioned before, there has few existing methods developed specifically to solve CSA miRNA-target association prediction problem. Therefore, we compared MTAGCN with three state-of-the-art approaches proposed to address other association prediction tasks in the computational biology.

- GCMDR [53] constructed a graph convolutional network based model to identify miRNA-drug resistance relationships.
- GATMDA [54] proposed a graph attention networks model with inductive matrix completion to predict human microbe-disease associations.
- GCNMDA [55] deployed a conditional random field on the graph convolution network to predict human microbe-drug associations.

**Fig. 4:**
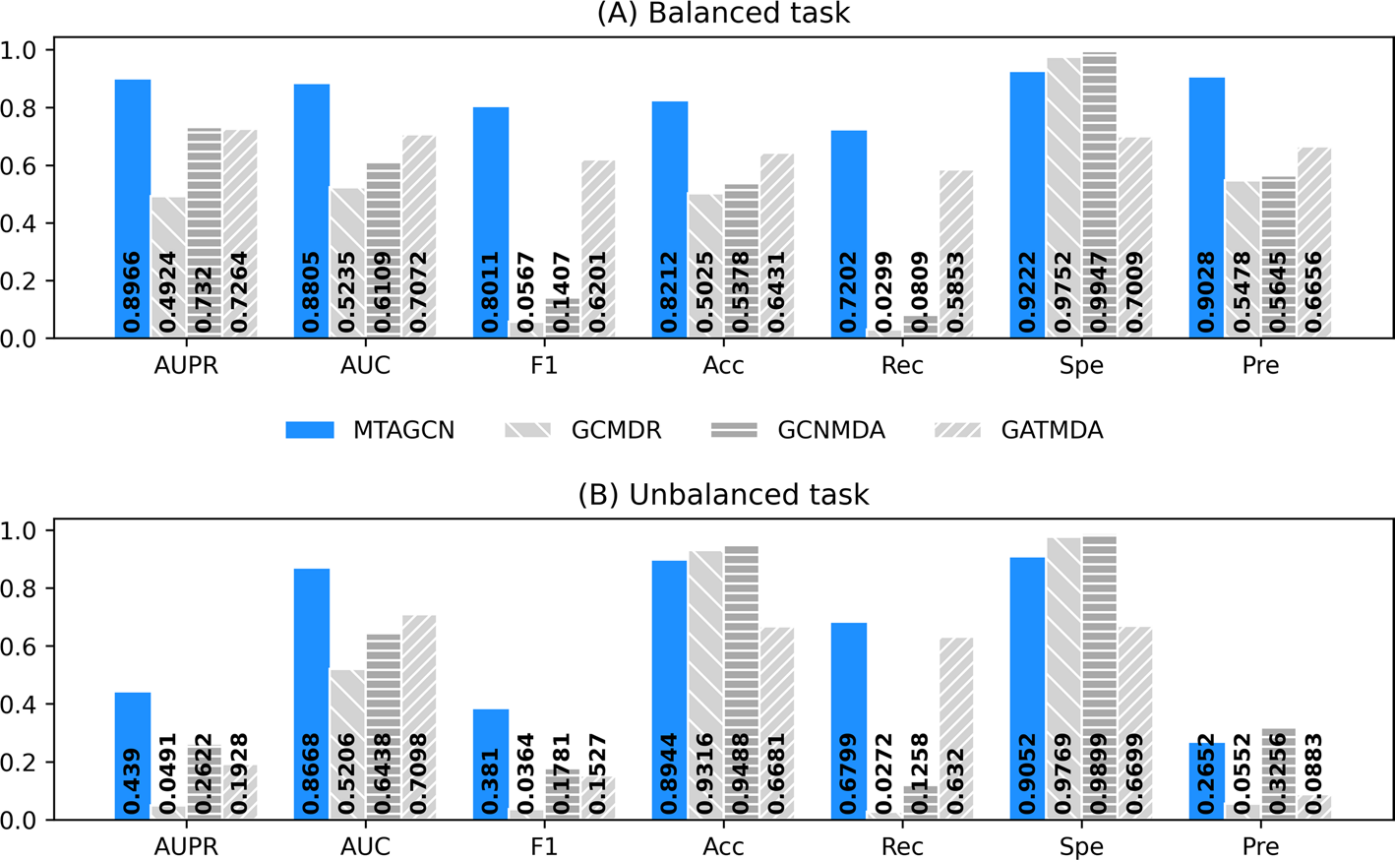
Performance comparison between MTAGCN and the state-of-the-art methods on (**A**) the balanced task and (**B**) the unbalanced task. F1, Acc, Rec, Spe and Pre represent the F1-score, accuracy, recall, specificity and precision, respectively

We compared them with our proposed model under the same experimental conditions, including balanced and unbalanced tasks. The results are shown in Fig. 4. We can observe that among all the methods under the balanced task in Fig. 4 (A), MTAGCN achieves the best performance. For the unbalanced task, although GCMDR, GCNMDA have slightly higher accuracy and specificity values than MTAGCN, overall, MTAGCN has better performance on the other metrics in Fig. 4 (B). As mentioned above, accuracy and specificity are greatly affected by data imbalance. In addition, MTAGCN outperformed all compared deep-learning methods in the most evaluation metrics. Furthermore, we would explain the reason why GCMDR obtained such low F1, recall and precision, finding that predicted scores of the true positive samples are almost close to 0. It is believed that the robustness of the GCMDR model is not good for CSA miRNA-target prediction problem.

### Parameter sensitivity

**Fig. 5:**
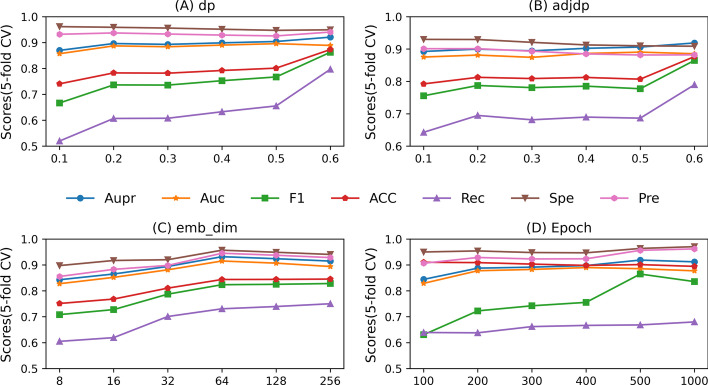
Parameter sensitivity under balanced task across node dropout (α), adjdp dropout (β), embedding dimensionality (*k*) and total training epoch (*T*). F1, Acc, Rec, Spe and Pre represent the F1-score, accuracy, recall, specificity and precision, respectively

There are several important parameters influence our model performance, such as the coarse-grained node dropout rate *α*, fine-grained edge dropout rate *β*, the embedding dimensionality *k* and the total training epoch *T*. In order to assess the parameter sensitivity, we evaluated the influences using five-fold CV for all parameters based on balanced task. The node dropout rate *α* plays an important role in our model. We ranged *α* from 0.1 to 0.6 with a step value of 0.1. As shown in Fig. 5, we can achieve the best performance when *α* = 0.6 and a small value of *α* is not good for the model performance. *β* is the regular dropout rate of the edge. We evaluated the performance of model by varying *β* from 0.1 to 0.6 with a step of 0.1. From Fig. 5, we could conclude that this parameter has a relatively slight influence on our model performance, which indicates that our model is robust against the regular dropout rate *β*. In addition, we used *k* to control the dimensionality of embeddings. In our experiment, we varied *k* from the range of {8, 16, 32, 64, 128, 256}. It can be observed that the best performance is achieved when *k* is 64 and the performance decreases if the value of *k* further increases in the Fig. 5. Lastly, we also considered the influence of total training epoch *T*. Results in Fig. 5 show that, our model produces the robust performances to the training epoch, which first slightly increases and then decreases, with epoch = 500 achieving the best performance.

### Case study

To verify the performance of the proposed model on CSA miRNA-target prediction task, we conducted case studies for CSA miRNA associated with targets. csn-MIR156j_5p is a conserved miRNA in the leaf and root degradomes of CSA, which plays an important role in organ/tissue-specific physiological and developmental process [7]. It has high expression levels with the functions of photosynthesis and transmembrane transport, regulating target CSA019508.1 and CSA015924.1 respectively. Some studies also proved that this miRNA could bind to the target CSA018458.1 to inhibit the secretion of resistance proteins [56]. csn-MIR319e_5p is a miRNA that influences the ATPase activity and ATP metabolic process related gene expression. For example, it can combine with the target TEA00633.1 and TEA000574.1 to reduce CSA respiration [31]. csn-MIR390b_3p is a conserved miRNA that relates to the structural constituent ribosome and oxygen-containing compound. Recent studies show that there is a close relationship between this miRNA and the CSA photosynthesis when targeting CSA024193.1 and CSA016339.1 [56]. However, huge challenges remain to reveal the mechanism of miRNA because of its functional complexity. Table 6 lists the results of the three case studies. It is obvious that they all show superior performances, demonstrating that the proposed MTAGCN model is capable of predicting the undiscovered potential miRNA-target associations for CSA miRNAs and targets.

**Table 6:** The summary of case studies for csn-MIR156j_5p, csn-MIR319e_5p and csn-miR390b_3p

CSA miRNA	AUPR	AUC	F1	Accuracy	Recall	Specificity	Precision
csn-MIR156j_5p	0.95536	0.9999	0.8333	0.9993	0.8333	0.9997	0.8333
csn-MIR319e_5p	0.95536	0.9999	0.7692	0.9990	0.8333	0.9993	0.7143
csn-miR390b_3p	0.96621	0.9998	0.8333	0.9986	0.9091	0.9990	0.7692

## Conclusion

In this paper, we proposed a novel deep learning framework, named MTAGCN, based on graph convolution network with layer attention for CSA miRNA-target association prediction. Compared with existing methods utilizing the topological graphs, MTAGCN integrates the graph information of the heterogeneous network built from CSA miRNA-target associations, CSA miRNA-miRNA similarity network and CSA target-target similarity network. Furthermore, MTAGCN adaptively combines embeddings at diverse convolution layers. Extensive experimental results demonstrate that MTAGCN outperforms the existing link/association prediction methods in predicting CSA miRNA-target associations.

However, although our model has good prediction performance, there is still room to enhance MTAGCN through further refinement. Due to the noise in the features extracted from similarity networks, our model is far from perfect, and the prediction results can be further improved. As a fast-growing research field, graph construction and multi-source feature fusion methods are boosting the model performance. For later versions of MTAGCN, we aim to further work closely with other study groups and develop the model on more experimentally verified data about CSA miRNA-target link associations.

## Supplementary Information

**Additional file 1**. Performance of MTAGCN based on different embeddings for the unbalanced task.

## Data Availability

The source code and processed data are freely available at https://github.com/haisonF/MTAGCN. The CSA miRNA information is available at the Supplementary Table 23 in Suo et al., https://ars.els-cdn.com/content/image/1-s2.0-S0888754320320188-mmc1.xlsx. The CSA target information is available online at http://teacon.wchoda.com.
