# Supplementary material for: MTAGCN: predicting miRNA-target associations in Camellia sinensis var. assamica through graph convolution neural network
Source: BMC Bioinformatics. 2022 Jul 11;23:271. doi: 10.1186/s12859-022-04819-3 (PMC9275082; doi:10.1186/s12859-022-04819-3)
Supplement: Supplementary file 1 — Additional file 1. Performance of MTAGCN based on different embeddings for the unbalanced task. [file 12859_2022_4819_MOESM1_ESM.docx]

Additional file 1**.** Performance of MTAGCN based on different embeddings for the unbalanced task.

| Performance  Models | AUPR | AUC | F1 | Accuracy | Recall | Specificity | Precision |
| --- | --- | --- | --- | --- | --- | --- | --- |
| MTAGCN | **0.7326** | **0.8744** | **0.6884** | 0.8923 | 0.7130 | 0.9282 | 0.6667 |
| MTAGCN-AVE | 0.7304 | 0.8258 | 0.4807 | 0.6358 | 0.3858 | 0.8857 | **0.7756** |
| MTAGCN-CON | 0.7139 | 0.8316 | 0.6778 | **0.9634** | **0.8005** | **0.9715** | 0.5911 |
| MTAGCN-L1 | 0.6814 | 0.8497 | 0.5120 | 0.9296 | 0.7583 | 0.9382 | 0.4892 |
| MTAGCN-L2 | 0.4630 | 0.8258 | 0.3812 | 0.8893 | 0.7121 | 0.8982 | 0.2602 |
| MTAGCN-L3 | 0.4414 | 0.8389 | 0.3078 | 0.8517 | 0.6921 | 0.8596 | 0.1979 |

Note: The maximum value of each metric is bold.
